# Psychometric Properties of Achievement Goal Constructs for Chinese Students

**DOI:** 10.3389/fpsyg.2020.531568

**Published:** 2020-10-26

**Authors:** Ningning Zhao, Yanfang Zhai, Xiaohan Chen, Meiling Li, Ping Li, Kunyu Ye, Hongbo Wen

**Affiliations:** ^1^School of Chinese Language and Literature, Beijing Normal University, Beijing, China; ^2^College of Education, Capital Normal University, Beijing, China; ^3^Collaborative Innovation Center of Assessment for Basic Education Quality, Beijing Normal University, Beijing, China; ^4^Beijing Yucai Middle School, Beijing, China

**Keywords:** Chinese, achievement goal structure, class, student, culture

## Abstract

In Chinese schools, classes are organized with special monitors and teachers contributing to the achievement goal structure for students. This study aimed to examine the psychometric properties of perception of teachers’ achievement goal structure constructs with 3,149 Chinese students from grades 3–8. The results showed that the internal consistencies of the whole scale and subscales were low to marginal. Eight models were examined to check the constructs of the achievement goal structure (mastery, performance, and performance avoidance). Two-factor structures proved to be the best fit. Additionally, a multilevel confirmatory factor analysis proved that the achievement goal structure existed at the same time in the class student levels. Our findings supported the hypothesis that achievement goal structures are different for students with different cultures, which implies that teaching approaches should be adapted in consideration of culturally distinct learning.

## Introduction

Motivation in learning contexts has been shown to be highly predictive of learning performance (Meece et al., 2006). In the past two decades, many studies have examined how students’ motivation interacts with learning contexts. The interaction with students’ motivation and teachers’ expectations in the classroom has been shown to affect students’ motivation and academic achievement (Good and Brophy, 2003). Students’ perceptions of teachers’ expectations have great influence on learning interest, persistence, and adaptation to learning strategies (Urdan, 2010). *Achievement goal theory* is an influential theory that focuses on the goal-directed behaviors of students (Dweck and Leggett, 1988; Elliot, 1997) and considers the interactions between students and other variables (teachers, peers, etc.). There were different arguments on the construct of the achievement goal model; for example, whether it is a 3 (task, self, and other) × 2 (approach and avoidance) model, a 2 (task and self) × 2 (approach and avoidance) model or a trichotomous model (Elliot, 1999; Pintrich, 2000; Elliot et al., 2011). Current research on achievement goals generally include three types of goals: mastery, performance approach, and performance avoidance. Moreover, it is important to develop valid instruments for students’ motivation under the achievement goal theory.

The Patterns of Adaptive Learning Scales (PALS) are one of the instruments that are developed under the achievement goal model. The PALS are used to investigate the relation between a learning environment and a student’s motivation, affect, and behavior. Although the PALS have been widely used, some problems with its usability remain.

The first is the applicability of the factor structure in different cultures. While empirical studies have supported different structures of the achievement goal model in different cultural contexts (David, 2014; Ning, 2018) and most revealed that there were three dimensions for the PALS (Alivernini et al., 2018), some studies in Japan showed that there were only two dimensions of mastery and performance goals for the PALS (Murayama and Elliot, 2009).

The second is that it is unclear as to whether students’ individual motivation can be aggregated to be represented at the classroom level. Recently, some studies argued that students with similar achievement are often aggregated to create a classroom level in America with students from grades 7–8 (Lam et al., 2015). However, the results have shown that only the performance-avoidance model is represented in the expected goal structure at the classroom level in America. Moreover, in different cultural contexts, there are different types of interaction between students and teachers. First, the collectivism aspect of Chinese culture emphasizes hard work, effort, and perseverance, which may have significant influence on learning goal orientations (Hau and Salili, 1996). Second, Chinese students have a special class structure in schools in compulsory education from grades 1–9. All students are in one class with a special monitor and one subject teacher. Thus, the teacher’s attitude on the achievement goal affects the whole class. Consequently, for different classes in China, there might be different kinds of achievement goals for the whole class.

However, limited studies on the construct of the achievement goal have been carried out in China. The present study explored whether students’ perception of teachers’ achievement goal structures in terms of the PALS can be applied in the Chinese context and whether this perception can be used as a measure of a class level’s motivational climate.

## Literature Review

This study explored the constructs of achievement goals theory by using PALS in Chinese context.

### Constructs of the Achievement Goals Theory

Different constructs of achievement goals have been developed in the past four decades. The original construct was dichotomous, with two types of goals identified: mastery goals and performance goals. *Mastery goals* stress the development of ability, competence, and skills, while *performance goals* focus more on how well or how poorly one behaves and demonstrates skill in the learning tasks compared to oneself or others (Dweck, 1986; Dweck and Leggett, 1988; Ames, 1992).

Later, a trichotomous model was developed, dividing the performance goals into *performance-approach goals* and *performance-avoidance goals* (Elliot and Harackiewicz, 1996). A performance-approach goals is focused on trying to outperform others. A performance-avoidance goal is focused on avoiding showing incompetence (Elliot and Harackiewicz, 1996). Later, mastery goals were also divided into approach and avoidance components, leading to a 2 × 2 model (Elliot, 1999; Pintrich, 2000). Master goals refer to a genuine desire to learn and master a task, while mastery-avoidance goals refer to wanting to avoid learning, thus leaving the task unfinished (Elliot, 1999; Pintrich, 2000).

Recently, the construct of achievement goals evolved into a 3 × 2 model based on the two valences of the trichotomous model, further defining three competences: *task*, *self*, and *other* (Elliot et al., 2011). Task-based goals show willingness to complete a task, whether it is to get the correct answer or understand an idea, and self-based goals lead to comparison with oneself in the past to see if competence improved. Other-based goals are interpersonal and involve evaluating oneself by comparison with others (Elliot et al., 2011). Thus, a 3 × 2 model was created with a *task-approach goal*, *task-avoidance goal*, *self-approach goal*, *self-avoidance goal*, *an other-approach goal*, and *an other-avoidance goal*.

### Cultural Context and Constructs of Achievement Goals

The constructs of achievement goals vary across different cultures (Hulleman et al., 2010). However, existing studies assume that the constructs of achievement goals are transferable between cultures and different groups.

Many empirical studies support the trichotomous model that comprises the mastery-approach, performance-approach, and performance-avoidance goals. Research in Hong Kong shows that the three achievement goals are validated and significantly correlated with learning achievement (Chan, 2010). A study in Turkey showed that the trichotomous achievement goal framework proved to be effective in middle school physical education (Agbuga and Xiang, 2008). On the other hand, results from a study in a rural school district of south-central Texas revealed that the trichotomous achievement goal model fit the data well and demonstrated satisfactory psychometric properties (Agbuga, 2009). Recently, another study compared the factor structures for grades 5–11 in Germany and Sweden (Hofverberg and Winberg, 2020). They found that only the mastery and performance goals were confirmed in Sweden, while the trichotomous achievement goal was proved in Germany (Hofverberg and Winberg, 2020). Hence, the aforementioned results indicate that the model is not freely transferable between countries.

Meanwhile, other studies have revealed that a 2 × 2 achievement goal model is the best fit in different cultures. In this context, a study on Turkish undergraduate students showed that the 2 × 2 achievement goal model was the best fit model (Agbuga, 2009). Another study by McInerney and Ali (2006) found that there was invariance across different cultural groups from the Australia, United States, Canada, Hong Kong, and Africa with four motivations of mastery, performance, social factors, and extrinsic factors. Moreover, a study using an American college students sample found that the 2 × 2 achievement goal model had a better fitting effect than the other three- or two-factor structures (Elliot and McGregor, 2001; Elliot and Murayama, 2008). The 2 × 2 achievement goal model was also found to have a better dimensional structure in Taiwan (Chiang et al., 2011) and Malaysia (Ganesan et al., 2014). Furthermore, Murayama et al. (2009) found that the achievement motivation model could be applied to students from different cultural backgrounds in the comparative analysis of students from Japan and Canada. However, another study using Chinese and Indonesian students as the sample found that the 2 × 2 achievement goals may not apply to East Asian cultures (Liem and Nie, 2008).

Additionally, the 3 × 2 achievement goal model has been validated using empirical studies with undergraduate students in Germany and the United States by confirmatory factor analysis (CFA; Elliot et al., 2011). A study in a Philippine sample also provided some support for the structural and predictive validity of the 3 × 2 model for task- and self-based achievement goals (David, 2014). Moreover, findings from CFAs provided strong support for the proposed structure of the 3 × 2 achievement goal model in Austrian college students (Lüftenegger et al., 2016) as well as in a secondary Spanish school (Méndez-Giménez et al., 2017, 2018). In addition, a study that used an Asian sample from Hongkong also revealed that the 3 × 2 model of achievement goals is the best fit model (Ning, 2018). Further, the achievement goal theory has also been applied to various other fields. For example, in e-learning, the 3 × 2 model was found to be a better fit than the trichotomous model based on a study with undergraduate students (Yang and Cao, 2013). Additionally, the 3 × 2 achievement goal model has also been tested among various professions. The model proved to be a good fit when studied in terms of French athletes and workers (Gillet et al., 2015; Mascret et al., 2015) as well as Singaporean athletes (Wang et al., 2017). Moreover, it has been proven that the model can be applied to teachers (Mascret et al., 2017).

To summarize, there are some cultural differences to be considered when using the achievement goal model (Wang et al., 2017). However, only limited studies can be found using the 3 × 2 model, 2 × 2 model, or trichotomous model in China. A CFA showed the 3 × 2 achievement goal model to have an excellent fit with undergraduate students in Hong Kong (Ning, 2018). Another study on Taiwan’s students showed that the 3 × 2 achievement goal model fits better than the dichotomy, trichotomy, or 2 × 2 model, but the elementary school students’ data did not fit as well as that of junior high school students (Wu, 2012). Furthermore, Wu (2012) explained that elementary school students probably could not discriminate goals. Hence, further studies with elementary school students in China is needed to test the model with a larger sample.

### Measures of Achievement Goals: PALS

The measures of students’ perceptions of teacher’s goals based on the achievement goal theory have been proven in different cultures. Among the studies on trichotomous goal framework, scales were developed to measure the three goals (Midgley et al., 2001; Finney et al., 2004). In this context, the PALS survey is one of the most popular instruments used to measure perceptions based on the achievement goal theory. The PALS survey on students’ perceptions of teacher’s goals consists of three subscales: a *teacher mastery goal*, *teacher performance-approach goal*, and *teacher performance-avoidance goal*.

Recently, there were some argumentations on the PALS goals’ constructs in different cultures (David, 2014; Ning, 2018). Most studies found that there were three dimensions for the PALS (Midgley et al., 2000; Alivernini et al., 2018). A study of Indonesian students’ test anxiety used the PALS to measure students’ perceptions and supported the validity of the scales (Dewi and Mangunsong, 2012). The validity and internal consistency were originally proven based on a study of four different samples of elementary and middle school students in the United States (Midgley et al., 1998). The general reliability for scales using the PALS has also been supported by studies with middle school students in the United States (Smart, 2014), students from grades 7 through 12 in China (Shi et al., 2001), and with grade 4 elementary students and college students in the United States (Ross et al., 2002). Moreover, in Turkey, the adaptive learning scale is still an effective tool (Parlak-Yilmaz and Çikrikçi-Demirtaşli, 2010). Similarly, in China, the experiment using Tianjin university students for the sample effectively verified three dimensions of the PALS (Ross et al., 2002). However, other studies in Japan and Sweden still showed that there were only two verifiable factors—mastery and performance goals—of the PALS (Murayama and Elliot, 2009). In this context, the researchers from the Sweden-based study stated that only two factors were proved because of varying cultures (Hofverberg and Winberg, 2020).

Additionally, some studies claimed that the motivation for individual students’ achievements can be aggregated to represent the classroom level (Lam et al., 2015). Miller and Murdock (2007) used the three-level hierarchical linear model to analyze students’ perceptions of goal structures and found that individual-level data could be aggregated to the classroom level (Miller and Murdock, 2007). The classroom achievement goal structure still has a unique effect after controlling for the predictive effect of individual achievement goals. Further, a study on grade 5 students in Singapore showed that there was a cross-level interaction between students’ achievement goal structures of the PALS and classroom achievement goal structures (Lau and Nie, 2008).

To summarize, the PALS has been proven to have high reliability and validity as an important instrument measuring achievement goals. However, limited studies have been conducted in the Chinese context. As previously shown, the culture, which includes aspects such as individualism and collectivism, might impact the constructs related to the PALS. In addition, there were some new trends for studies on the meaning of aggregated measurement, which might vary across cultures.

## Materials and Methods

### Research Questions

The present study aimed to examine the factor structure of the students’ perception of teachers’ goals with a sample of Chinese students by using PALS under the achievement goal theory. It included two questions:

(1) Are the structures of the mastery, performance, and performance-avoidance goals in the PALS fit for Chinese students?
(2) Can the measures at the individual level be used as the class-level measure by using the students’ perceptions of teachers’ goals section of PALS?

### Participants

A total of 3,149 students in 152 classes from grades 3–8 participated in this study. By a specific grade, the student demographics were as follows: 608 students (19.3%) were from 30 third-grade classes, 615 students (19.5%) were from 32 fourth-grade classes, 664 students (21.1%) were from 33 fifth-grade classes, 613 students (19.5%) were from 30 sixth-grade classes, 297 students (9.4%) were from 13 seventh-grade classes, and 352 students (11.2%) were from 14 eighth-grade classes. The average class size was about 21 students. In total, there were 1,426 (45.3%) girls. Students were diverse across a wide range of variables and nationally representative. Three economic development levels were distinguished, based on data about the regional gross domestic product (GDP). As a result, distribution of pupils in these regions were as follows: 39.3% in higher GDP provinces, 19.8% in middle GDP provinces, and 40.9% in low GDP provinces, respectively. The demographic characteristics of participants can be found in Table 1.

**TABLE 1:** Weighted demographic characteristics of participants.

**Characteristic**	**Weighted%**
**Gender**	
Female	45.6
**Grade**	
3	19.3
4	19.5
5	21.1
6	19.5
7	9.4
8	11.2
**Area**	
Higher GDP	39.3
Middle GDP	19.8
Lower GDP	40.9

### Measures and Perceptions of Teachers’ Goals

The perception of teachers’ goal structures included 12 items—consistent with the original questionnaire (PALS) (Midgley et al., 2000)—that were assessed via student self-reports. Each of the items was rated using a scale from “1” (never) to “6” (always) instead of the 5-point Likert scale to avoid having one response as the middle ground. The teachers’ goal structures consisted of three dimensions: *mastery goal*, *performance-approach goal*, and *performance-avoidance goal*. This factor structure was supported by nine school districts in three Midwestern states (Midgley et al., 2000) with 3,149 participants and a 100% completion rate with the scale. The reliability coefficient for the overall scale was *a* = 0.79. The reliability coefficient for subscales including mastery, performance approach, and performance avoidance was *a* = 0.73, *a* = 0.65, *a* = 0.71, respectively. Item wording and descriptive statistics data are shown in Table 2, including the item-level valid responses, alpha coefficients, means, and standard deviations (SD).

**TABLE 2:** Item-level descriptive data.

	***M***	***SD***
Mastery (*α* = 0.73)	4.23	1.06
My teacher thinks mistakes are okay as long as we are learning.	3.60	1.71
My teacher wants us to understand our work, not just memorize it.	4.40	1.68
My teacher really wants us to enjoy learning new things.	4.64	1.38
My teacher recognizes us for trying hard.	4.17	1.40
My teacher gives us time to really explore and understand new ideas.	4.32	1.46
Performance approach (*α* = 0.65)	4.22	1.20
My teacher points out those students who get good grades as an example to all of us.	4.87	1.35
My teacher lets us know which students get the highest scores on a test	4.28	1.60
My teacher tells us how we compare to other students.	3.51	1.72
Performance avoidance (*α* = 0.71)	4.35	1.15
My teacher tells us that it is important that we do not look stupid in class.	3.10	1.70
My teacher says that showing others that we are not bad at class work should be our goal.	4.22	1.55
My teacher tells us it is important to join in discussions and answer questions so it does not look like we cannot do the work.	3.29	1.72
My teacher tells us it is important to answer questions in class, so it does not look like we cannot do the work.	2.92	1.65

### Procedure

The questionnaires were one part of the nationally supported project for children’s learning performance. Permission for data collection was granted from the students and teachers, and all information about individual students will be kept confidential.

### Data Analysis

First, CFAs were conducted to examine the eight competing structures using Mplus 7.4 (Muthén and Muthén, 1998–2015). The eight competing models included the following: (M1–M3), single-factor models; (M4–M6), two correlated-factors models each with two of three factors; (M7), a three correlated-factors model (12 items); and (M8), a one second-order factor with three first-order factors model (12 items). The method of maximum likelihood was used in the present study.

Second, multilevel confirmatory factor analyses (MCFAs) were used to investigate whether the perception of teachers’ goals could be used as measures of class-level motivational climates. There were 12 models used for the MCFAs: (M1–M5), a single-factor MCFA including two combined models with a *mastery–performance goal* and *performance–performance avoidance goal*; (M6–M10), a two-factor MCFA, with models 6a–10a with freely estimated loading and model 6a–10b with fixed equal loading between students and class level; (M11), a three-factor MCFA; and (M12–M13), mixed models with different factors in class and student levels. The data can be get accessed by Supplementary Table 1.

## Results

### Descriptive Statistics

Table 2 provides the mean and standard deviation for each item and for the three achievement goal structure scales. The mean was 4.23 (*SD* = 1.06) for mastery and 4.22 (*SD* = 1.20) and 4.35 (*SD* = 1.15) for performance approach and performance-avoidance goal, respectively.

First, a CFA was used to examine the possible models of the classroom goal questionnaires, and the results are presented in Table 3. The single factor models all fit well. The results for two-factor models also showed good fit for the mastery and performance goal and the mastery and performance-avoidance goal. However, in model 6, the two-factor models with performance and performance-avoidance goals showed marginal fit; the three-factor model of the achievement-goal model (model 7) also showed only a marginal model fit.

**TABLE 3:** Confirmatory factor analysis (CFA) for single-level factor structure.

	**χ^2^**	**df**	**CFI**	**TLI**	**RMSEA**	**SRMR**	**BIC**	**AIC**
Model 1: Mas CFA	69.267	4	0.98	0.949	0.072	0.023	54,687.388	54,590.51
Model 2: Per CFA	44.388	1	0.969	0.907	0.117	0.057	33,666.732	33,618.293
Model 3: Avo CFA	15.585	3	0.994	0.988	0.036	0.023	46,258.171	46,191.568
Model 4: Mas & Per CFA	249.165	17	0.957	0.929	0.066	0.033	87,743.051	87,579.57
Model 5: Mas & Avo CFA	315.134	26	0.951	0.932	0.059	0.037	100,638.434	100,468.899
Model 6: Per & Avo CFA	248.377	11	0.945	0.895	0.083	0.034	79,306.653	79,161.337
Model 7: Mas & Per & Avo CFA	822.981	46	0.91	0.871	0.073	0.052	133,424.53	133,158.118
Model 8: Mas & Per & Avo High order	876.421	48	0.904	0.868	0.074	0.053	133,461.861	133,207.557

*Mas, mastery goal; Per, performance goal; Avo, performance avoidance goal.*

In addition, the high-order factor model was also analyzed to determine if there were high-order factors in addition to the master goal, the performance approaching goal, and the performance-avoidance goal. The high order model assumed that there is a higher-order latent variable, which can include master goal, performance-approach goal, and performance-avoidance goal.

### Measures of Individual Student Constructs as the Class Level Climate

An MCFA on achievement goals was conducted to explore the internal factor structure at both the student and class levels. Additionally, the reliability and validity of student reports of classroom goal structure were analyzed within an MCFA with the trichotomous model. As can be seen from Table 4, the results show that the scale has better measurement indicators when the scales are aggregated to the class level. At the individual level, only the mastery goal factor’s composite reliability (CR) value was above 0.7, other two dimensions’ CR values were <0.7. Meanwhile, the internal consistency of construct index is low. The average variance extracted (AVE) is below 0.36, which shows that the reliability and convergence validity of latent variables are lower than the requirements of measurement. However, at the class level, the lowest CR value was 0.77, and the lowest AVE was 0.55. Moreover, intraclass correlation (ICC) were also calculated. ICC1 were between 0.16 and 0.21, which indicates the reliability of the score within the group, while ICC2 were between 0.80 and 0.85, which indicates the reliability of the mean group score. These results showed that the reliability and validity of the scale meet the requirements for the aggregated analysis (Geldhof et al., 2014). In summary, the results show that the scale has high internal consistency, construct validity, and aggregate validity.

**TABLE 4:** Composite reliability (CR), average variance extracted (AVE), and intraclass correlation (ICC) for multilevel confirmatory factor analysis (MCFA).

	**Within**		**Between**		**ICC1**	**ICC2**
	**CR**	**AVE**	**CR**	**AVE**		
1. Mastery goal	0.71	0.34	0.87	0.58	0.20	0.84
2. Performance-approach goal	0.59	0.33	0.77	0.55	0.16	0.80
3. Performance-avoidance goal	0.69	0.36	0.85	0.59	0.21	0.85
Total	0.51	0.34	0.73	0.58	–	–

First, single-factor MCFAs were performed; the model fitting indexes were compared with the cutoff value with good model fitting. In Model 1 of the mastery goal structure, standardized factor loadings ranged from 0.387 to 0.766 at the within level and from 0.543 to 0.929 at the class level (see Table 5). The overall model fit was acceptable (see Table 5). At the student level, the model fit was acceptable; however, the fit of the model at the class level was just above the critical value of 0.08. For Model 2, for the performance goal, the standard factor load ranged from 0.489 to 0.779 at the individual level and from 0.318 to 0.917 at the class level. The overall model fit was acceptable. At the individual level, the model fit for the performance-approach goal was acceptable, while the model fit had a slightly high at the class level. In Model 3 of the performance-avoidance goal, the standardized factor loading ranged from 0.560 to 0.618 at the within level and from 0.587 to 0.981 at the between level. The fit of the model was acceptable at both the student and class levels.

**TABLE 5:** Multilevel confirmatory factor analysis results.

**Model**	**Standard factor load**		**Model fitting**							
	**Within**	**Between**	**×2 (df)**	**AIC**	**BIC**	**CFI**	**TLI**	**RMSEA**	**SRMRw**	**SRMRb**
**Single-factor model**										
1. Mastery goal	0.387–0.766	0.543–0.929	67.063* (9)	53,626.674	53,784.100	0.980	0.955	0.044	0.019	0.089
2. Performance-approach goal	0.489–0.779	0.318–0.917	14.137* (1)	32,737.073	32,821.841	0.991	0.944	0.065	0.001	0.093
3. Performance-avoidance goal	0.560–0.618	0.587–0.981	9.424* (4)	45,598.399	45,719.496	0.997	0.992	0.021	0.008	0.050
4. Performance-approach goal combined with Performance-avoidance goal	0.377–0.647	0.346–0.828	317.765* (25)	77,801.856	78,031.939	0.931	0.883	0.061	0.035	0.188
5. Mastery goal combined with performance-approach goal	0.144–0.736	0.357–0.940	229.264* (37)	85,843.966	86,104.324	0.961	0.941	0.041	0.023	0.168
**Two-factor models**										
6a. Mastery goal + performance-approach goal			191.074*(36)	85,807.775	86,074.188	0.968	0.951	0.037	0.022	0.125
Mastery goal	0.392–0.734	0.542–0.950								
Performance-approach goal	0.312–0.963	0.276–0.968								
6b. Mastery goal + performance-approach goal: loadings constrained to be equal			315.101* (43)	85,917.803	86,141.832	0.945	0.928	0.045	0.024	0.299
Mastery goal	0.396–0.713	0.699–0.996								
Performance-approach goal	0.347–0.797	0.618–1.022								
7a. Mastery goal + performance-avoidance goal			294.533* (53)	98,926.767	99,205.289	0.955	0.939	0.038	0.031	0.134
Mastery goal	0.388–0.693	0.544–0.942								
Performance-avoidance goal	0.548–0.660	0.708–0.973								
7b. Mastery goal + performance-avoidance goal: loadings constrained to be equal			352.828* (58)	98,975.062	99,223.31	0.945	0.932	0.04	0.029	0.364
Mastery goal	0.398–0.675	0.708–0.987								
Performance-avoidance goal	0.526–0.641	0.888–0.970								
8a. Performance-approach goal + performance-avoidance goal			234.944* (24)	77,721.034	77,957.173	0.950	0.912	0.053	0.028	0.165
Performance-approach goal	0.503–0.805	0.332–0.893								
Performance-avoidance goal	0.523–0.681	0.573–0.970								
8b. Performance-approach goal + performance-avoidance goal: loadings constrained to be equal			330.420* (28)	77,808.511	78,020.430	0.928	0.892	0.059	0.028	0.401
Performance-approach goal	0.452–0.774	0.787–0.928								
Performance-avoidance goal	0.486–0.684	0.890–0.957								
9. Performance-approach goal combined with performance-avoidance goal + mastery goal			775.068* (102)	130,821.614	131,221.234	0.916	0.891	0.046	0.043	0.179
Performance-approach goal combined with performance-avoidance goal	0.371–0.648	0.284–0.880								
Mastery goal	0.391–0.691	0.536–0.947								
10. Mastery goal combined with performance-approach goal + performance-avoidance goal			795.601* (101)	130,844.147	131,249.821	0.913	0.887	0.047	0.055	0.173
Mastery goal combined with performance-approach goal	0.259–0.701	0.340–0.954								
Performance-avoidance goal	0.538–0.671	0.586–0.965								
**Three-factor models**										
11. Mastery goal + performance-approach goal + performance-avoidance goal			883.051* (101)	130,931.597	131,337.272	0.902	0.873	0.050	0.048	0.158
Mastery goal	0.393–0.693	0.480–0.972								
Performance-approach goal	0.487–0.665	0.488–0.874								
Performance-avoidance goal	0.532–0.669	0.596–0.968								

In previous studies, some researchers claimed that the performance and performance-avoid goals referred to the same dimension. Thus, a single-factor model, including all performance-approach and performance-avoidance items, was conducted in Model 4. In Model 4, the standardized factor loading ranged from 0.377 to 0.647 at the within level and from 0.346 to 0.828 at the between level. The overall model fit was acceptable with a slightly low Tucker–Lewis index (TLI). The fit of the model was acceptable at both the individual and the class levels.

Additionally, the mastery and performance-approach goals were combined as a whole factor (Model 5). The standard factor load ranged from 0.144 to 0.736 at the within level and from 0.357 to 0.940 at the between level. The overall model fit was acceptable. At the individual level, the model fit was acceptable, while the model fit was high at the classroom level.

Next, multilevel two-factor models were conducted from Models 6a–10. In Model 6a, two dimensions of achievement goals (mastery and performance-approach goals) were examined. The standard factor loading for the mastery goal at the individual level ranged from 0.392 to 0.734 and from 0.542 to 0.950 at the class level. Standard factor loading for the performance-approach goal at the individual level ranged from 0.312 to 0.936 and from 0.276 to 0.968 at the class level (see Table 5). The overall model fit was acceptable, while the individual level model fit was acceptable, and the class level model fit was marginal. To test that the coefficient load of each layer was equal, a further MCFA model (Model 6b) was estimated with the load of the first layer and the second layer limited to being equal. The Bayesian information criterion (BIC) index (86,141.832) of the constraint model (equal load) was larger than that of the free estimation model (BIC = 86,074.188), indicating that the overall hypothesis of equal loadings should be rejected; that is, Model 6a is more suitable.

For Model 7a, with two factors of mastery and performance-avoidance goals, it was found that the standard factor load for mastery goal ranged from 0.388 to 0.693 at the individual level and from 0.544 to 0.942 at the class level. The standard factor load for performance-avoid goal ranged from 0.548 to 0.660 at the individual level and from 0.708 to 0.973 at the class level. The overall model fit was acceptable. Meanwhile, the model fit was marginal Standardized Root Mean Residual (SRMR) (0.134) at the class level. In order to test that the coefficient load of each layer was equal, the MCFA model (Model 7b) was estimated with the loads of the first and second layers limited to being equal. The BIC index of the constraint model (equal load) was larger than that of the free estimation model, indicating that the overall hypothesis of equal loadings should be rejected; that is, Model 7a is more suitable.

For the performance-approach and performance-avoidance goals (Model 8a), the standardized factor loading ranged from 0.503 to 0.805 at the individual level and from 0.332 to 0.893 at the between level. The overall model fit for avoidance goal was acceptable. At the individual level, the fit of the model was also acceptable, indicating that performance-approach and performance-avoidance goals were two different latent constructs. However, the model fit at the class level had a slight fit. To test that the coefficient load of each layer was equal, the MCFA model (Model 8b) was estimated with the loads of the first and second layers limited to being equal. The BIC index of the constraint model (equal load) was larger than that of the free estimation model, indicating that the overall hypothesis of equal loadings should be rejected; that is, Model 8a was more suitable.

Based on Model 4, an MCFA was conducted with the performance-approach and performance-avoidance goals combined as one factor and the mastery goal as another factor. The results showed that the standard factor load for mastery goal ranged from 0.391 to 0.691 at the within level and from 0.536 to 0.947 at the between level. The standard factor load for the single factor (performance-approach and performance-avoidance goals) ranged from 0.371 to 0.648 at the individual level and from 0.284 to 0.880 at the class level. The overall model fit was acceptable except for a slightly low TLI (0.891). At the individual level, the model fit was acceptable, while the between-classroom level model fit was poor with the SRMR at 0.179.

Similarly, an MCFA was conducted with the combination of mastery and performance-approach goals as one factor and the performance-avoidance goal as the second factor in Model 10. The results showed that the standard factor load for performance-avoid goal ranged from 0.538 to 0.671 at the within level and from 0.586 to 0.965 at the between level. The standard factor load for the single factor (mastery and performance-approach goals) ranged from 0.259 to 0.701 at the individual level and from 0.340 to 0.954 at the class level. The overall model fit was acceptable except for the slightly low TLI (0.887). At the individual level, the model fit was acceptable, while the model fit at the class level was poor with the SRMR at 0.173.

Finally, MCFAs on the trichotomous achievement goal framework (mastery goal + performance-approach goal + performance-avoid goal) was conducted in Model 11. The finding (Model 11) showed that the overall model fit for the trichotomous achievement goal was acceptable except for the slightly low TLI (0.873) but only at individual level; the trichotomous achievement goal framework was acceptable, while the model fit was poor (SRMR 0.158) at the class level.

## Discussion

This study intended to address two persistent questions relating to the PALS by examining grade 3–8 students’ perceptions of teachers’ achievement goals. Findings from this study offer empirical evidence that helps reinforce prior proposals that there are two dimensions of mastery and performance-avoid goals in the Chinese context that is collectivistic (Hofverberg and Winberg, 2020). Additionally, the MCFA results showed that, within the Chinese context, students can recognize teachers’ achievement goals. However, students’ perceptions of the mastery goal varied across individuals. Thus, the PALS can be used to measure class-level achievement goals in China because of the class system.

### Two Dimensions of Mastery and Performance as the Achievement Goal Theory in the Chinese Context

Our first research question concerned what achievement goal factor structure of the PALS is the most appropriate in the Chinese context. Results from the single-, two-, and three-factor CFAs suggest that the approach factors of mastery and performance goals can be measured with the PALS in the Chinese context. Regarding the achievement goal theory, the trichotomous model was defined as follows: *task*, *self*, and *other* (Elliot et al., 2011). However, in the present study, the three-factor model, two factors with performance and performance avoidance, showed only a marginal fit for Chinese students. This result implies that the *self* and *other* might have the same meaning for Chinese students. Chinese culture emphasizes collectivism (Hofstede, 1980); thus, achievement goals are often described as being for the benefit of the group (study group or family) rather than the individual. In Confucian heritage culture, Chinese students view learning as being two sides: it is a process of moral striving for self-improvement but also performance with an extrinsic reward (Li, 2002). Thus, the mastery and the performance models fit well when we consider the two sides of the achievement goal. In the present study, the two factor models of mastery–performance goal or mastery–performance-avoidance goal showed a better fit. This implied that the performance and performance-avoidance goals both work in the Chinese context. However, the performance and performance avoidance were not the same in Chinese context as the two-factor models of these goals were not good. This result is different from that of the previous study in Sweden (Hofverberg and Winberg, 2020). Previous achievement goal theory studies have argued that there are ways that performance-approach goals can combine with mastery goals to promote optimal motivation, emphasizing the importance of separating approach from avoidance strivings (Harackiewicz et al., 2002). In further studies, performance-avoidance goals should be explored in order to identify the different relationships with the other two factors.

### Mastery and Performance as Measures of Individual and Class Motivation Climates in China

The other research question focused on whether mastery, performance, and performance-avoidance subscales of the PALS in the student level can represent the class level. The results of multilevel CFAs revealed that all the single multilevel factor model fits were acceptable. In addition, they imply that students’ measures of the mastery goal, performance goal, and performance-avoidance goal could be used as measures for the class level. The results are in line with a previous study in America (Lam et al., 2015). In that study, only performance-avoidance goals were found to be acceptable. However, in the Chinese context, two factors of mastery and performance goals were slightly acceptable, which implies that class plays an important role in students’ motivation development when compared to American approaches to class structures. Additionally, the students’ perceptions of teachers’ mastery and performance goals may vary across students.

The results with regards to the trichotomous model in Table 4 also have some interesting implications. Only the mastery goal has a higher CR in both the individual and class levels. However, the CR of performance and performance-avoidance goals were accepted only in the class level. This implies that these goals might be aggregated in the class level. Students in one class could have more similar perceptions of teachers’ goals for higher performance. Nevertheless, students in one class might have different perceptions as to the motivation for tasks. The teacher may be only one factor that can improve students’ motivation, and students’ motivations for a task might come from themselves. These results also confirm the two dimensions of the achievement goal. The MCFA results in Table 5 also have some interesting implications. The two-factor MCFAs showed that Model 6a was better than 7a with the two dimensions of mastery and performance goal having a marginal fit. The combination of the performance-approach and mastery factors is reflected at both student and class levels in China. This indicates that Chinese students can also identify the teachers’ goals at the student and class levels. The meaning of this for education and learning may be related to the two sides of the learning coin.

## Conclusion, Limitations, and Future Research

To summarize, the present study aims to explore the internal factor structure in the context of Chinese culture. The results support the single- and two-factor models of the achievement goal structure with a good fit and the three-factor models with a marginal fit via the examination of seven competing structures. Next, MCFAs were used to examine whether the achievement goal at the individual level can be used in the class-level climate. The results support the two-factor models with two dimensions of mastery and performance at the individual student level. It also showed that the measures of the two factors with performance and mastery goals at the individual level can be used as class level motivation in China. Additionally, the performance-avoidance factor also works as both the individual- and class-level single factor, although it cannot be added to three-factor models with the other two factors.

This study contributes to the understanding of the constructs of the achievement goal theory. First, the results support the two-factor model of the mastery and performance goals in the Chinese context, in which the meanings of mastery and performance are similar for Chinese students; therefore, performance and mastery were combined as the achievement goal. Second, it also implies that the mastery and performance goals could be measured together at the student and class levels. However, each item has a different meaning at each different level because of the changes in loading at the different levels.

This study also has some limitations. First, the construct may be different for students in primary school from for those in secondary school. Further studies could divide the participant samples into two different grade-level groups to discern any differences. Second, the present study only explored the construct of achievement goal studies in China and did not compare the different cultures in the CFA. Comparison studies could be conducted in the future. Third, the present study only examined the construct of *perception* of teachers’ goals, which is only one part of the PALS. Other parts of the PALS could be analyzed in further studies. Finally, the present study only explored the reliability of the PALS in China and its multilevel application but did not examine the validity of the PALS in China. In the future, more studies should be carried out for that.

## Data Availability Statement

The datasets generated for this study are available on request to the corresponding author.

## Ethics Statement

The studies involving human participants were reviewed and approved by the examination form of experimental ethics of Department of Psychology of Beijing Normal University. Written informed consent to participate in this study was provided by the participants’ legal guardian/next of kin.

## Author Contributions

NZ and HW conceived and designed the study, collected the data, and helped perform the analysis with constructive discussions. YZ, ML, XC, PL, and KY performed the data analyses and wrote the manuscript. All authors contributed to the article and approved the submitted version.

## Conflict of Interest

The authors declare that the research was conducted in the absence of any commercial or financial relationships that could be construed as a potential conflict of interest.

## Supplementary Material

Supplementary Data Sheet 1 Data for the article.
